# Association between non-high-density lipoprotein cholesterol to high-density lipoprotein cholesterol ratio (NHHR) and kidney stone: evidence from NHANES 2007–2018

**DOI:** 10.1186/s12889-024-19265-4

**Published:** 2024-07-08

**Authors:** Tao Chen, Yu Cheng, Zheng Song, Gan Zhang, Tao Zeng, Haichao Chao

**Affiliations:** 1 Department of Urology, the second Affiliated Hospital of Nanchang University, Nanchang, China; 2 Medical college of Nanchang University, Nanchang, China

**Keywords:** NHHR, Kidney stones, Lipid ratio, RCS, Cross-sectional study

## Abstract

**Background:**

As an innovative lipid parameter, NHHR (the ratio of non-high-density lipoprotein cholesterol to high-density lipoprotein cholesterol) can serve as a valuable tool for assessing cardiovascular disease risk. Nevertheless, the relationship between NHHR and the risk of kidney stones remains unexplored.

**Methods:**

A cross-sectional survey utilized data from the National Health and Population Survey (NHANES) database in the United States spanning from 2007 to 2018. Distinct statistical analyses were applied, including weighted logistic regression, stratified and interaction analysis and restricted cubic spline curve (RCS) models, to examine the correlation between NHHR and the incidence of kidney stones.

**Results:**

This analysis encompassed 24,664 participants, with 9.63% reporting incidents of kidney stones. Following multivariate logistic regression and comprehensive adjustments, participants in NHHR quartile 4 (OR 1.34; 95% CI 1.12, 1.60, *P* < 0.01) exhibited a significantly increased risk of kidney stones compared to those in NHHR quartile 1 (Q1). The RCS result further illustrated a non-linear correlation between NHHR and the incidence of kidney stones. The result of subgroup analysis manifested that participants without diabetes had a higher risk of kidney stones when measured high NHHR levels compared those with diabetes (*p for interaction* < 0.05).

**Conclusion:**

Elevated NHHR levels were found to be associated with an increased risk of kidney stones. Based on these findings, NHHR appears to be a promising predictive indicator for the occurrence of kidney stones.

**Supplementary Information:**

The online version contains supplementary material available at 10.1186/s12889-024-19265-4.

## Background

Over the last few decades, kidney stone incidence has increased rapidly and steadily worldwide. For example, the occurrence rate in the United States increased from 3.2% in the 1980s to 9.6% at present [1, 2]. Symptomatic kidney stones are more prevalent in men, with the peak incidence occurring between the ages of 40 and 60 for males and at 50 for females [3, 4]. Contributing to this rising trend are factors such as obesity, diabetes (DM), metabolic syndrome, and dietary patterns [5–7]. The escalating medical costs and substantial societal burden associated with kidney stones have garnered significant attention.

Presently, numerous studies have delved into the intricate relationship between lipid metabolism and the formation of kidney stones. For a cross-sectional study involving 20,972 participants aged 19 and above, a higher triglyceride glucose index was identified as being associated with an increased likelihood of kidney stones [8]. Furthermore, a preliminary retrospective study found that people with high triglyceride (TG) and total cholesterol (TC) had a markedly increased risk of uric acid stones [9]. According to large cohort studies, individuals with hyperlipidemia have a significantly lower risk of developing new kidney stones than people without statin therapy [10].

The ratio of non-high-density lipoprotein cholesterol (non-HDL-C) to HDL-C (NHHR) is a recently developed comprehensive indicator used to assess atherogenic lipid mass spectrometry, offering a comprehensive insight into both anti-atherosclerosis and atherogenic lipid particles [11]. Previous research has underscored NHHR’s superior diagnostic performance in estimating the likelihood of insulin resistance, metabolic syndrome, and cerebrovascular disease when compared to standard lipid parameters [12–14]. Hence, investigating the correlation between NHHR and kidney stones holds promise in offering insightful information about how lipid metabolism and urinary stones interact. This exploration may catalyze further research on preventive strategies and intervention measures.

## Methods

### Study population

**Fig. 1:**
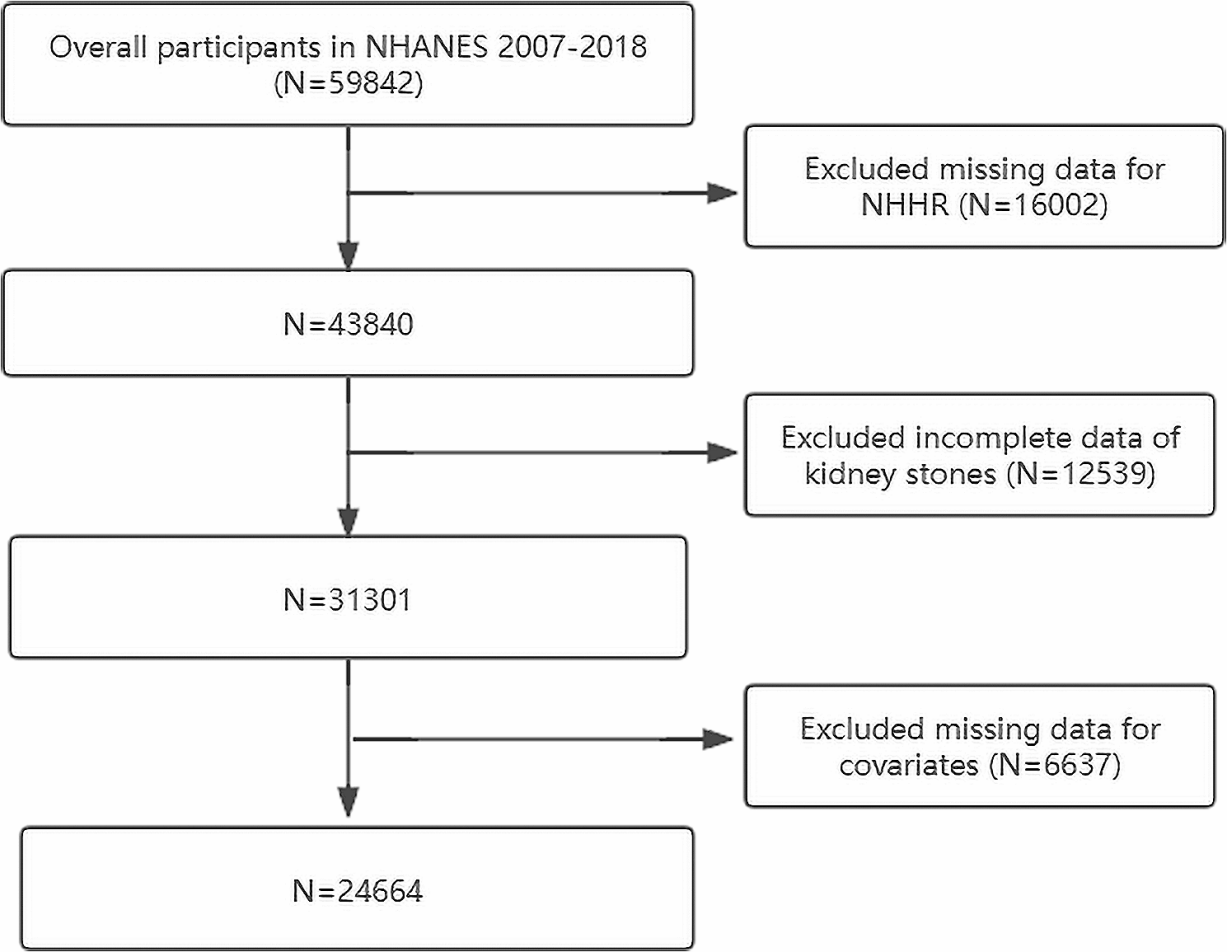
Flowchart displaying the selection of participants

This study adopted a cross-sectional design based on the NHANES dataset. Between 2007 and 2018, a total of 59,842 individuals were recruited. Exclusion criteria comprised: (a) missing data on kidney stones; (b) incomplete NHHR data; (c) missing covariate data. Ultimately, 24,664 participants met the criteria for inclusion in the complete case analysis (Fig. 1).

### NHHR exposure assessment

NHHR, serving as the independent variable in exposure assessment, was calculated following methods established in prior studies, specifically utilizing the Non HDL-C/HDL-C ratio [15]. By deducting HDL-C from total cholesterol (TC), non HDL-C was yield. The automated biochemical analyzer performed enzymatic testing to assess TC and HDL-C levels.

### Determination of kidney stones

In this analysis, details regarding the history of kidney stones were gathered using a targeted questionnaire focused on kidney conditions. A proficient interviewer, well-trained for this purpose, identified cases of kidney stones through personal interviews. The criterion for defining a case of kidney stones was straightforward: Individuals were deemed to have a history of kidney stones if they answered “yes” to the inquiry. Considering that the NHANES database only contains data on kidney stone recurrence during the period of 2007–2014, we only analyzed the recurrence of kidney stones during this time period. How many times have you had kidney stones? (NHANES 2007–2014). Participants who had at least two stones were classified as those with recurrent stone formation.

### Ascertainment of covariables

Covariables were chosen as potential confounding factors to enhance the model’s reliability in establishing associations. Following insights from previously published literature [8, 16–18], The covariates that were chosen included age, sex (male or female), race (Non-Hispanic black, non-Hispanic white, Hispanic/Mexican, and other races), education, and poverty income ratio (< 1.3, 1.3–3.5, and > 3.5); lifestyle factors, such as body mass index (BMI) (< 25, 25-29.99, or > = 30 kg/m2), alcohol drinking (Never, Mild, Moderate and Heavy), and smoking (Never, Former and Current); and chronic illnesses (No or Yes).

### Statistical analysis

In this analysis, appropriate NHANES sampling weight was applied. Weighted student t-tests or chi-square tests were utilized to assess intergroup differences for continuous and categorical variables, respectively. Three distinct logistic regression models were utilized to investigate the independent relationship between NHHR and kidney stones. Model 1 did not account for covariates; Model 2 did so based on age, sex, education, race and poverty income ratio; and Model 3 did so along with alcohol consumption, physical activity, BMI, smoking status, diabetes, and hypertension. A cubic spline curve was applied to estimate the dose-response relationship, providing a thorough assessment of the nonlinear correlation between NHHR and kidney stones. Subgroup analysis was carried out to assess the robustness of the correlation. R software (version 4.2.0) was applied for all analyses, and statistical significance was defined as *P* < 0.05.

## Results

### Baseline characteristics of participants

This study encompassed 24,664 participants, with the kidney stone incidence of 9.63%. The average age of participants was 47.44 ± 0.26 years. Compared with participant without kidney stones, those with kidney stones were more likely to be older, male, Non-Hispanic white, with a larger BMI, less exercise, smoking and drinking, as well as suffering from hypertension and diabetes (*P* < 0.001). Table 1 displays specific baseline characteristics. In addition, as shown in Table 2, we analyzed the baseline characteristics of participants with recurrent kidney stones. Patients with recurrent kidney stones are more likely to be Non-Hispanic white and suffer from diabetes, in which the level of NHHR is higher.

**Table 1:** Baseline characteristics of participants by kidney stones from the NHANES, 2007–2018

Characteristics	Total (*N* = 24,664)	Normal (*n* = 22,288)	Kidney stones (*n* = 2376)	*P* value
Age, years, mean (SD)	47.44 ± 0.26	46.81 ± 0.27	53.06 ± 0.37	**< 0.0001**
Gender, n (%)				**< 0.0001**
Male	12,265 (49.28)	10,927 (48.63)	1338 (55.19)	
Female	12,399 (50.72)	11,361 (51.37)	1038 (44.81)	
Race, n (%)				**< 0.0001**
Non-Hispanic White	10,818 (69.09)	9480 (68.04)	1338 (78.52)	
Non-Hispanic Black	4968 (10.15)	4665 (10.67)	303 (5.48)	
Mexican American	3632 (8.13)	3330 (8.38)	302 (5.90)	
Other Hispanic	2470 (5.36)	2214 (5.42)	256 (4.85)	
Other race	2776 (7.28)	2599 (7.50)	177 (5.24)	
Education, n (%)				0.7
High school grad or equivalent	9703 (44.58)	8827 (44.62)	876 (44.17)	
Less than high school	5644 (14.58)	5070 (14.50)	574 (15.25)	
Some college or above	9317 (40.84)	8391 (40.87)	926 (40.58)	
PIR, n (%)				0.24
< 1.3	7789 (21.09)	7044 (21.26)	745 (19.51)	
1.3–3.5	3346 (10.61)	3025 (10.54)	321 (11.24)	
> 3.5	13,529 (68.31)	12,219 (68.20)	1310 (69.25)	
BMI, n (%)				**< 0.0001**
< 25	6977 (29.16)	6518 (30.24)	459 (19.36)	
25-29.99	8114 (32.83)	7322 (32.88)	792 (32.40)	
≥ 30	9573 (38.01)	8448 (36.87)	1125 (48.25)	
Moderate recreational activities, n (%)				**0.003**
No	14,485 (53.05)	12,970 (52.59)	1515 (57.28)	
Yes	101,079 (46.95)	9318 (47.41)	861 (42.72)	
Smoking status, n (%)				**< 0.0001**
Never	13,575 (55.54)	12,427 (56.18)	1148 (49.75)	
Former	6014 (24.94)	5272 (24.35)	742 (30.21)	
Current	5075 (19.52)	4589 (19.46)	486 (20.05)	
Alcohol drinking, n (%)				**< 0.0001**
Never	3413 (10.44)	3100 (10.48)	313 (10.14)	
Former	3858 (13.66)	3351 (12.13)	507 (17.39)	
Mild	8445 (37.32)	7574 (36.98)	871 (40.41)	
Moderate	3885 (17.88)	3578 (18.09)	307 (16.02)	
Heavy	5063 (21.07)	4685 (22.33)	378 (16.03)	
Hypertension, n (%)				**< 0.0001**
No	14,106 (62.08)	13,073 (63.73)	1033 (47.32)	
Yes	10,558 (37.92)	9215 (36.27)	1343 (52.68)	
Diabetes, n (%)				**< 0.0001**
No	19,949 (85.57)	18,285 (86.73)	1664 (75.14)	
Yes	4715 (14.43)	4003 (13.27)	712 (24.86)	

*BMI* body mass index, *RIP* ratio of family income to poverty

**Table 2:** Baseline characteristics of participants by recurrent kidney stones from the NHANES, 2007–2014

Characteristics	Total (*N* = 1589)	Non-recurrent kidney stones (*N* = 1077)	Recurrent kidney stones (*N* = 512)	*P* value
Age, years, mean (SD)	53.32(0.41)	53.56(0.57)	52.88(0.70)	0.49
Gender, n (%)				0.2
Female	685(44.41)	487(45.87)	198(41.67)	
Male	904(55.59)	590(54.13)	314(58.33)	
Race, n (%)				< 0.001
Black	196(5.25)	157(6.63)	39(2.68)	
Mexican American	184(5.52)	142(6.66)	42(3.38)	
Other Hispanic	161(4.40)	96(3.99)	65(5.16)	
Other race	90(4.40)	72(4.91)	18(3.46)	
White	958(80.42)	610(77.80)	348(85.33)	
Education, n (%)				0.07
High school grad or equivalent	565(40.72)	371(38.10)	194(45.62)	
Less than high school	408(17.12)	286(17.85)	122(15.77)	
Some college or above	616(42.15)	420(44.05)	196(38.61)	
PIR, n (%)				0.24
< 1.3	522(21.00)	355(21.62)	167(19.85)	
1.3–3.5	206(11.83)	139(10.87)	67(13.61)	
> 3.5	861(67.17)	583(67.50)	278(66.55)	
BMI, n (%)				0.16
< 25	312(20.36)	222(21.80)	90(17.66)	
25-29.99	548(32.75)	376(33.40)	172(31.53)	
≥ 30	729(46.90)	479(44.80)	250(50.81)	
Moderate recreational activities, n (%)				0.29
No	1029(60.11)	697(61.34)	332(57.82)	
Yes	560(39.89)	380(38.66)	180(42.18)	
Smoking status, n (%)				0.13
former	509(29.33)	338(30.04)	171(28.02)	
never	766(50.24)	542(51.45)	224(47.98)	
now	314(20.42)	197(18.51)	117(24.00)	
Alcohol drinking, n (%)				0.65
former	402(21.52)	261(20.03)	141(24.28)	
heavy	248(14.95)	171(15.42)	77(14.08)	
mild	556(38.91)	367(39.07)	189(38.60)	
moderate	178(13.61)	123(14.23)	55(12.44)	
never	205(11.01)	155(11.24)	50(10.60)	
Hypertension, n (%)				0.28
no	667(45.80)	448(44.59)	219(48.06)	
yes	922(54.20)	629(55.41)	293(51.94)	
Diabetes, n (%)				0.01
No	1118(75.29)	766(77.18)	352(71.76)	
Yes	471(24.71)	311(22.82)	160(28.24)	
NHHR	3.25(0.05)	3.18(0.06)	3.38(0.07)	0.02

### The association between NHHR and kidney stones

The association between NHHR and kidney stones was exhibited in Table 3. Using quartile 1 as the reference, all three models revealed a positive correlation between NHHR and kidney stones at the Q4 level [Model 1: OR (95% CI) 1.81 (1.54, 2.13); Model 2: OR (95% CI) 1.71 (1.46, 2.01); Model 3: OR (95% CI) 1.34 (1.12, 1.60)]. However, in the fully adjusted model, we did not find any association between NHHR levels and kidney stone recurrence (Supplementary Table 1). In addition, to compare NHHR with the presence of dyslipidemia, we utilized the history of dyslipidemia as a research variable to explore the impact of dyslipidemia on the occurrence of kidney stones. As shown in Supplementary Table 2, our results showed that after full adjustment, the history of dyslipidemia may have no significant impact on the occurrence of kidney stones.

**Table 3:** Relationship between NHHR and kidney stones in the logistic regression models from the NHANES, 2007–2018

	Model 1			Model 2			Model 3	
	OR (95%CI)	*P* value		OR (95% CI)	*P* value		OR (95% CI)	*P* value
NHHR								
Q1 (0.28–1.93]	Ref			Ref			Ref	
Q2 (1.93–2.68]	1.44 (1.21,1.70)	**< 0.0001**		1.40 (1.18,1.66)	**< 0.001**		1.27 (1.06,1.52)	**0.01**
Q3 (2.68–3.66]	1.67 (1.37,2.03)	**< 0.0001**		1.59 (1.31,1.93)	**< 0.0001**		1.35 (1.10,1.65)	**0.004**
Q4(3.66–26.66]	1.81 (1.54,2.13)	**< 0.0001**		1.71 (1.46,2.01)	**< 0.0001**		1.34 (1.12,1.60)	**0.002**
*P* for trend	**< 0.0001**			**< 0.001**			**0.002**	

Model 1: Adjusted for no covariates

Model 2: Adjusted for age, race, education, gender, and *RIP (*ratio of family income to poverty)

Model 3: Adjust for the variables in Model 2 plus BMI (body mass index), moderate recreational activity, smoking, alcohol drinking, hypertension, diabetes

*OR* odd ratio

### A nonlinear correlation between NHHR and kidney stones

**Fig. 2:**
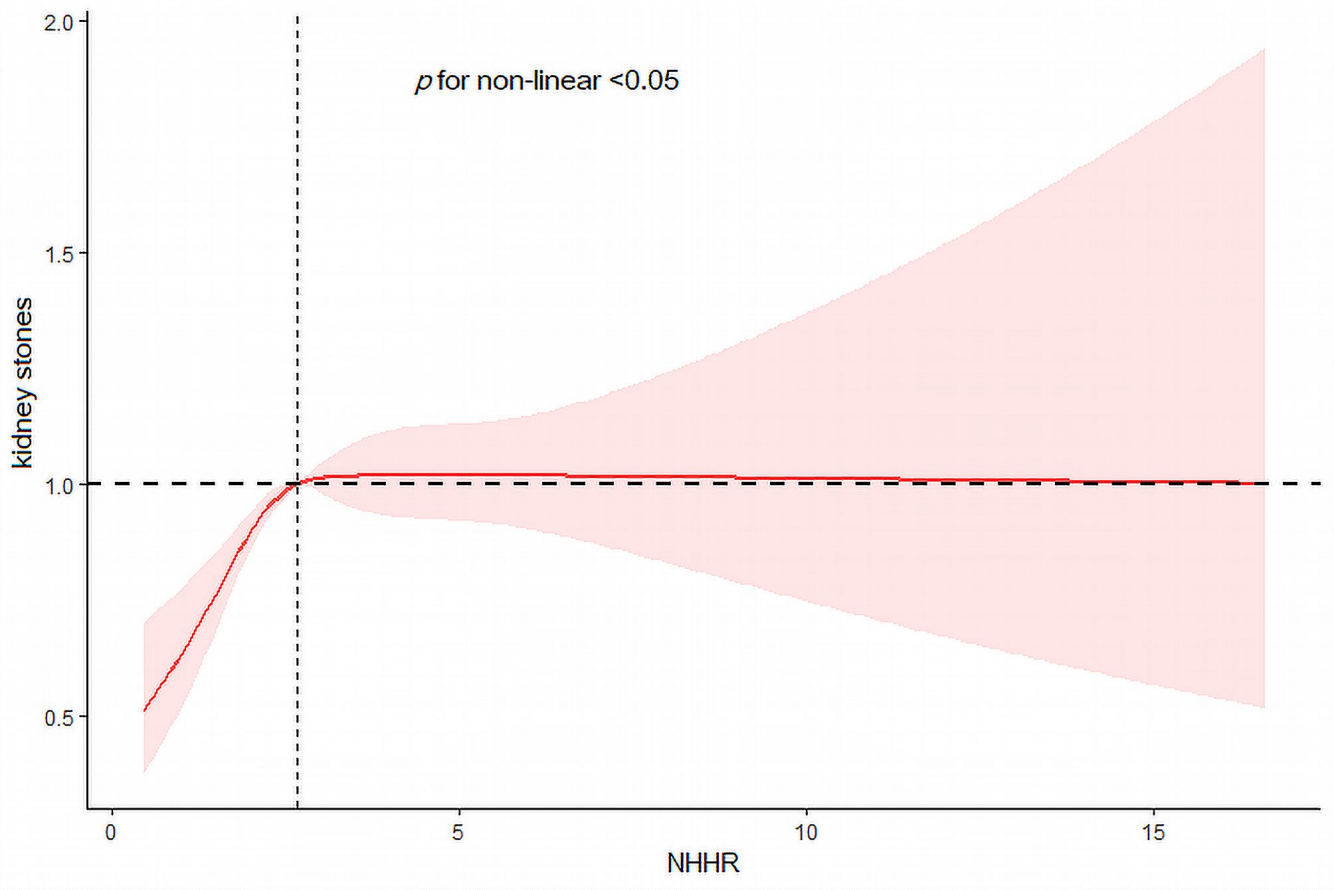
Restricted cubic spline of the association between NHHR levels and kidney stone disease adjusted for all covariates

In order to delved deeper into the non-linear relationship between NHHR and kidney stones, restricted cubic spline curves were employed (Fig. 2). The result suggested that the risk of kidney stones experienced a sharp increase in the early stages with the rise of NHHR, eventually stabilizing in the later stages (non-linear *P* < 0.05).

### Subgroup analysis

Subgroup analysis on all covariates were conducted, whose results manifested that participants without diabetes had a higher risk of kidney stones when measured high NHHR levels compared those with diabetes (Table 4). We found that in the group with BMI < 25 and no diabetes, the OR in the fourth quarter was lower than that in the second group. Considering the close correlation between NHHR levels and metabolic syndrome, we further conducted subgroup analysis based on the presence or absence of metabolic syndrome, As shown in Supplementary Table 3, we found that in patients without metabolic syndrome, NHHR indicators have a stronger impact on the occurrence of kidney stones.

**Table 4:** Results of subgroup analysis ^*a*^

Subgroup	Quartiles of NHHR				*P* for interaction
	Q1	Q2	Q3	Q4	
Age (years)					0.06
< 60	1	1.16 (0.89,1.53)	1.19 (0.90,1.56)	1.36 (1.07,1.73)*	
≥ 60	1	1.39 (1.11,1.75)*	1.58 (1.20,2.08)**	1.15 (0.87,1.52)	
Race, n (%)					0.44
Non-Hispanic White	1	1.26 (1.01,1.58)*	1.40 (1.09,1.82)*	1.35 (1.07,1.70)*	
Non-Hispanic Black	1	1.37 (0.95,1.99)	1.09 (0.74,1.60)	0.94 (0.56,1.55)	
Mexican American	1	0.96 (0.62,1.49)	0.99 (0.65,1.52)	1.00 (0.63,1.59)	
Other Hispanic	1	1.67 (1.04,2.71)*	1.23 (0.72,2.11)	1.56 (0.99,2.44)*	
Other race	1	1.29 (0.62, 2.70)	1.40 (0.69,2.82)	1.73 (0.83, 3.80)	
Education, n (%)					0.42
High school grad or equivalent	1	1.22 (0.91,1.64)	1.31 (0.93,1.83)	1.39 (1.04,1.84)*	
Less than high school	1	1.38 (0.95,2.01)	1.91 (1.44,2.53)**	1.33 (0.93,1.83)	
Some college or above	1	1.31 (0.93,1.80)	1.23 (0.87,1.72)	1.32 (0.99,1.78)	
PIR, n (%)					0.58
< 1.3	1	1.16 (0.86,1.58)	1.30 (1.00,1.62)*	1.22 (0.94,1.57)	
1.3–3.5	1	1.76 (1.05,2.97)*	1.61 (0.94,2.78)	1.81 (1.04,3.15)*	
>3.5	1	1.23 (0.99,1.54)	1.33 (1.01,1.78)*	1.32 (1.03,1.78)*	
BMI, n (%)					0.38
< 25	1	1.60 (1.21,2.12)**	1.20 (0.82,1.77)	1.15 (0.69,1.87)	
25-29.99	1	1.12 (0.80,1.56)	1.27 (0.96,1.68)	1.21 (0.90,1.61)	
≥ 30	1	1.20 (0.85,1.71)	1.42 (0.99,2.03)	1.42 (1.02,1.96)*	
Recreational activities, n (%)					0.94
No	1	1.28 (1.03,1.58)*	1.32 (1.07,1.66)*	1.36 (1.07,1.71)*	
Yes	1	1.26 (0.97,1.65)	1.39 (1.05,1.84)*	1.31 (0.96,1.79)	
alcohol.drinking, n (%)					0.30
Never	1	1.36 (0.83,2.25)	1.93 (1.13,3.30)*	1.91 (1.24,2.95)**	
Former	1	1.15 (0.76,1.73)	1.14 (0.76,1.71)	1.21 (0.86,1.70)	
Mild	1	1.12 (0.83,1.48)	1.50 (1.09, 2.06)	1.35 (0.99,1.85)*	
Moderate	1	1.46 (0.91,2.36)	1.22 (0.75,1.98)	1.32 (0.76,2.31)	
Heavy	1	1.59 (1.10,2.30)*	1.03 (0.71,1.49)	1.19 (0.79,1.79)	
Hypertension, n (%)					0.33
No	1	1.27 (0.99,1.63)	1.21 (0.92,1.60)	1.38 (1.06,1.81)*	
Yes	1	1.23 (0.95,1.59)	1.41 (1.07,1.84)*	1.20 (0.93,1.58)**	
Diabetes, n (%)					0.03
No	1	1.36 (1.10,1.68)**	1.38 (1.08,1.77)*	1.30 (1.02,1.58)*	
Yes	1	0.96 (0.72,1.27)	1.17 (0.85,1.62)	1.41 (1.05,1.89)*	

*BMI* body mass index, *RIP* ratio of family income to poverty

^*a*^ Adjusted for all potential confounding factors including age, race, education, sex, and *RIP (*ratio of family income to poverty), BMI (body mass index), recreational activity, smoking, hypertension, diabetes. The model was not adjusted for the factor itself in each stratification

*< 0.05; **< 0.01

## Discussion

In this cross-sectional study involving 24,664 adults, our results revealed that a higher NHHR index is independently related to an increased likelihood of kidney stones. Our speculation is that NHHR can function as a predictive factor for the information of kidney stones, and regulating lipid levels determined by NHHR may contribute to reducing the incidence of kidney stones.

NHHR, a recently combined index reflecting the lipid composition of atherosclerosis, surpasses traditional lipid parameters in evaluating the degree of atherosclerosis [19, 20]. Notably, NHHR has demonstrated excellent predictive power in various studies. Kwok et al. proposed that, in comparison to other lipid indicators, NHHR exhibits stronger predictive ability for non-alcoholic fatty liver disease (NAFLD) [21]. Additionally, Lin et al. identified NHHR as a reliable indicator for evaluating insulin resistance [22]. As a superior indicator of lipid-related disease risk, our study represents the first exploration of the correlation between the NHHR index and kidney stones. Previous research has investigated the relationship between kidney stones and various lipid-related factors. According to research by Fibio et al., those who have high levels of triglycerides (TG) and total cholesterol (TC) have a markedly increased risk of developing uric acid stones [9]. Cohen et al. Believed that statin consumption can prevent kidney stones from forming, which implies that statins’ anti-inflammatory and antioxidant qualities can lower the risk of kidney stones [10]. Moreover, a large cross-sectional study confirmed that the risk of kidney stone onset and recurrence is higher in individuals with a higher TyG index [8]. These findings indirectly support a positive correlation between NHHR levels and the occurrence of kidney stones, introducing new lipid features for studying the relationship between lipid features and kidney stones.

Several reasons may account for the observed association between NHHR and kidney stones. Obesity appears to be a proven risk factor for kidney stone, although we adjusted for BMI as a factor in our analysis. Obesity is frequently associated with metabolic syndrome, which includes insulin resistance, hypertension, and lipid abnormalities [23–25]. NHHR, as an indicator of lipid abnormalities, often exhibits high levels in obese populations. This correlation arises because obesity is associated with elevated levels of low-density lipoprotein cholesterol (LDL-C) and very low-density lipoprotein cholesterol (VLDL-C), while high-density lipoprotein cholesterol (HDL-C) levels often decrease, leading to an increase in NHHR [26]. Patients with obesity often experience disturbances in lipid metabolism, leading to changes in the distribution and utilization of fat in the body. These changes not only increase LDL-C and VLDL-C levels but also decrease HDL-C levels, further increasing NHHR [27]. Moreover, obesity is often accompanied by chronic inflammation and oxidative stress, both of which may lead to abnormal lipid metabolism and the progression of atherosclerosis, thus elevating NHHR. These inflammatory factors and oxidative stress can damage the vascular endothelium and increase the risk of atherosclerosis [28, 29]. The relationship between obesity and kidney stones is complex, and NHHR may be an important mediator in this context. Obesity leads to an increase in NHHR through various mechanisms, such as lipid metabolism disorders, chronic inflammation, oxidative stress, among others, thereby increasing the risk of kidney stones. In addition, obesity itself is closely related to metabolic syndrome, which is an important risk factor for the formation of kidney stones. Previous studies have highlighted the involvement of multiple inflammatory processes in the formation of kidney stones, suggesting that inflammation plays a role in their occurrence and development [30, 31]. Obese mice have demonstrated increased renal crystal formation in response to metabolically induced inflammation [32]. Another study using an ethylene glycol mouse model with high oxaluria found that atorvastatin treatment reduced the retention of renal crystals, supporting the notion that inflammation resulting from metabolic changes may contribute to kidney stone formation [33]. Additionally, according to the result of RNA sequencing of renal papillae bearing Randall plaques, impaired lipid metabolism may play a major role in the development of these stones [34, 35]. Furthermore, It has been demonstrated that kidney stone risk is positively correlated with diabetes, obesity, hypertension, and dyslipidemia. These conditions frequently coexist with dyslipidemia [36, 37]. Nevertheless, the substantial association between NHHR and kidney stones remains in this study even after adjusting for diabetes, BMI, and hypertension. This implies that NHHR might have a distinct role in the information of this disease.

Our research exhibits several strengths. Firstly, it was based on NHANES data and utilized appropriate sample weights for analysis. Secondly, the adjustment for confounding covariates enhanced the reliability of results. However, it’s crucial to acknowledge the limitations of our research. Firstly, the diagnosis of kidney stones relies on personal interviews, introducing potential biases from the interview process. Secondly, the NHANES database does not provide specific information on the types of kidney stones, and therefore, we cannot investigate the impact of NHHR levels on specific types of kidney stones. Thirdly, the cross-sectional study design prevents establishing a causal relationship between the NHHR index and kidney stones. Despite adjusting for multiple confounding factors, the influence of other unaccounted variables cannot be completely eliminated. Therefore, caution is warranted in interpreting the research results.

## Conclusion

The observed association indicating that a higher NHHR index is linked to an increased likelihood of developing kidney stones suggests significant implications for kidney stone prevention. This finding underscores the potential importance of monitoring and managing NHHR levels as part of strategies aimed at preventing kidney stones.

### Electronic supplementary material

Below is the link to the electronic supplementary material.

Supplementary Material 1

## Data Availability

The dataset analyzed during this study can be found in NHANES: https://www/cdc/gov/nchs/nhanes/.

## Abbreviations

HDL: C High-density lipoprotein cholesterol
Non: HDL-C Non-high-density lipoprotein cholesterol
NHHR Non: HDL-C and HDL-C
TC: Cholesterol
BMI: Body mass index

## References

[1] Mao W, Hu Q, Chen S, Chen Y, Luo M, Zhang Z, Geng J, Wu J, Xu B, Chen M (2021). Polyfluoroalkyl chemicals and the risk of kidney stones in US adults: a population-based study. Ecotoxicol Environ Saf.

[2] Scales CD, Smith AC, Hanley JM, Saigal CS (2012). Prevalence of kidney stones in the United States. Eur Urol.

[3] Kittanamongkolchai W, Vaughan LE, Enders FT, Dhondup T, Mehta RA, Krambeck AE, McCollough CH, Vrtiska TJ, Lieske JC, Rule AD. The Changing Incidence and Presentation of Urinary Stones Over 3 Decades. Mayo Clinic proceedings. 2018;93(3):291–299. 10.1016/j.mayocp.2017.11.018 PMC5849397 29452705

[4] Lieske JC, de la Peña LS, Slezak JM, Bergstralh EJ, Leibson CL, Ho KL, Gettman MT (2006). Renal stone epidemiology in Rochester, Minnesota: an update. Kidney Int.

[5] Geiss LS, Wang J, Cheng YJ, Thompson TJ, Barker L, Li Y, Albright AL, Gregg EW (2014). Prevalence and incidence trends for diagnosed diabetes among adults aged 20 to 79 years, United States, 1980–2012. JAMA.

[6] Meyer KA, Harnack LJ, Luepker RV, Zhou X, Jacobs DR, Steffen LM (2013). Twenty-two-year population trends in sodium and potassium consumption: the Minnesota Heart Survey. J Am Heart Association.

[7] Bleich SN, Wang YC, Wang Y, Gortmaker SL (2009). Increasing consumption of sugar-sweetened beverages among US adults: 1988–1994 to 1999–2004. Am J Clin Nutr.

[8] Qin Z, Zhao J, Geng J, Chang K, Liao R, Su B (2021). Higher triglyceride-glucose index is Associated with increased likelihood of kidney stones. Front Endocrinol.

[9] Torricelli FC, De SK, Gebreselassie S, Li I, Sarkissian C, Monga M (2014). Dyslipidemia and kidney stone risk. J Urol.

[10] Cohen AJ, Adamsky MA, Nottingham CU, Pruitt J, Lapin B, Wang CH, Park S (2019). Impact of statin intake on kidney stone formation. Urology.

[11] Zhu L, Lu Z, Zhu L, Ouyang X, Yang Y, He W, Feng Y, Yi F, Song Y (2015). Lipoprotein ratios are better than conventional lipid parameters in predicting coronary heart disease in Chinese Han people. Kardiologia Polska.

[12] Iannuzzi A, Giallauria F, Gentile M, Rubba P, Covetti G, Bresciani A, Aliberti E, Cuomo G, Panico C, Tripaldella M et al. Association between Non-HDL-C/HDL-C Ratio and Carotid Intima-Media Thickness in Post-Menopausal Women. Journal of clinical medicine. 2021;11(1). 10.3390/jcm11010078 PMC8745439 35011818

[13] Kim SW, Jee JH, Kim HJ, Jin SM, Suh S, Bae JC, Kim SW, Chung JH, Min YK, Lee MS (2013). Non-HDL-cholesterol/HDL-cholesterol is a better predictor of metabolic syndrome and insulin resistance than apolipoprotein B/apolipoprotein A1. Int J Cardiol.

[14] Yang S, Zhong J, Ye M, Miao L, Lu G, Xu C, Xue Z, Zhou X (2020). Association between the non-HDL-cholesterol to HDL-cholesterol ratio and non-alcoholic fatty liver disease in Chinese children and adolescents: a large single-center cross-sectional study. Lipids Health Dis.

[15] Wang A, Li Y, Zhou L, Liu K, Li S, Zong C, Song B, Gao Y, Li Y, Tian C (2022). Non-HDL-C/HDL-C ratio is associated with carotid plaque stability in general population: a cross-sectional study. Front Neurol.

[16] Di XP, Gao XS, Xiang LY, Wei X (2023). The association of dietary intake of riboflavin and thiamine with kidney stone: a cross-sectional survey of NHANES 2007–2018. BMC Public Health.

[17] Di X, Liu S, Xiang L, Jin X (2023). Association between the systemic immune-inflammation index and kidney stone: a cross-sectional study of NHANES 2007–2018. Front Immunol.

[18] Song W, Hu H, Ni J, Zhang H, Zhang H, Yang G, Wang Y, Zhang Y, Peng B (2023). The relationship between ethylene oxide levels in hemoglobin and the prevalence of kidney stones in US adults: an exposure-response analysis from NHANES 2013–2016. Environ Sci Pollut Res Int.

[19] Zhao W, Gong W, Wu N, Li Y, Ye K, Lu B, Zhang Z, Qu S, Li Y, Yang Y (2014). Association of lipid profiles and the ratios with arterial stiffness in middle-aged and elderly Chinese. Lipids Health Dis.

[20] Sheng G, Liu D, Kuang M, Zhong Y, Zhang S, Zou Y (2022). Utility of Non-high-density Lipoprotein Cholesterol to high-density lipoprotein cholesterol ratio in evaluating Incident Diabetes Risk. Diabetes Metabolic Syndrome Obesity: Targets Therapy.

[21] Kwok RM, Torres DM, Harrison SA (2013). Vitamin D and nonalcoholic fatty liver disease (NAFLD): is it more than just an association?. Hepatology (Baltimore MD).

[22] Lin D, Qi Y, Huang C, Wu M, Wang C, Li F, Yang C, Yan L, Ren M, Sun K (2018). Associations of lipid parameters with insulin resistance and diabetes: a population-based study. Clin Nutr.

[23] Mir FA, Ullah E, Mall R, Iskandarani A, Samra TA, Cyprian F, Parray A, Alkasem M, Abdalhakam I, Farooq F et al. Dysregulated metabolic pathways in subjects with obesity and metabolic syndrome. Int J Mol Sci 2022;23(17). 10.3390/ijms23179821 PMC9456113 36077214

[24] Litwin M, Kułaga Z (2021). Obesity, metabolic syndrome, and primary hypertension. Pediatr Nephrol.

[25] Blüher M. Metabolically healthy obesity. Endocr Rev 2020;41(3). 10.1210/endrev/bnaa004 PMC7098708 32128581

[26] Huang Y, Gao L, Cheng H, Wang X, Dong H, Yan Y, Zhao X, Liu J, Shan X, Mi J (2023). Difference of glucose and lipid metabolism abnormalities and body fat between the Chinese and USA teenagers. J Global Health.

[27] Klop B, Elte JW, Cabezas MC (2013). Dyslipidemia in obesity: mechanisms and potential targets. Nutrients.

[28] Gianfrancesco MA, Paquot N, Piette J, Legrand-Poels S (2018). Lipid bilayer stress in obesity-linked inflammatory and metabolic disorders. Biochem Pharmacol.

[29] Lee MC, Hsu YJ, Sung HC, Wen YT, Wei L, Huang CC. Low aerobic capacity accelerates lipid Accumulation and metabolic abnormalities caused by High-Fat Diet-Induced obesity in Postpartum mice. Nutrients 2022;14(18). 10.3390/nu14183746 PMC9502809 36145123

[30] Kumar P, Yang Z, Lever JM, Chávez MD, Fatima H, Crossman DK, Maynard CL, George JF, Mitchell T (2022). Hydroxyproline stimulates inflammation and reprograms macrophage signaling in a rat kidney stone model. Biochim et Biophys acta Mol Basis Disease.

[31] Khan SR, Canales BK, Dominguez-Gutierrez PR (2021). Randall’s plaque and calcium oxalate stone formation: role for immunity and inflammation. Nat Rev Nephrol.

[32] Taguchi K, Okada A, Hamamoto S, Iwatsuki S, Naiki T, Ando R, Mizuno K, Tozawa K, Kohri K, Yasui T (2015). Proinflammatory and Metabolic Changes Facilitate Renal Crystal Deposition in an obese mouse model of metabolic syndrome. J Urol.

[33] Tsujihata M, Momohara C, Yoshioka I, Tsujimura A, Nonomura N, Okuyama A (2008). Atorvastatin inhibits renal crystal retention in a rat stone forming model. J Urol.

[34] Taguchi K, Hamamoto S, Okada A, Unno R, Kamisawa H, Naiki T, Ando R, Mizuno K, Kawai N, Tozawa K (2017). Genome-wide gene expression profiling of Randall’s plaques in Calcium Oxalate Stone formers. J Am Soc Nephrology: JASN.

[35] Taguchi K, Chen L, Usawachintachit M, Hamamoto S, Kang M, Sugino T, Unno R, Tzou DT, Sherer BA, Okada A (2020). Fatty acid-binding protein 4 downregulation drives calcification in the development of kidney stone disease. Kidney Int.

[36] Yuan S, Larsson SC (2021). Assessing causal associations of obesity and diabetes with kidney stones using mendelian randomization analysis. Mol Genet Metab.

[37] Lin BB, Huang RH, Lin BL, Hong YK, Lin ME, He XJ (2020). Associations between nephrolithiasis and diabetes mellitus, hypertension and gallstones: a meta-analysis of cohort studies. Nephrol (Carlton Vic).

