# Supplementary material for: Association between non-high-density lipoprotein cholesterol to high-density lipoprotein cholesterol ratio (NHHR) and kidney stone: evidence from NHANES 2007–2018
Source: BMC Public Health. 2024 Jul 8;24:1818. doi: 10.1186/s12889-024-19265-4 (PMC11232242; doi:10.1186/s12889-024-19265-4)
Supplement: Supplementary file 1 — Supplementary Material 1 [file 12889_2024_19265_MOESM1_ESM.docx]

**Table S1.** Relationship between NHHR and Recurrent kidney stones in the logistic regression models from the NHANES, 2007–2014

|  | Model 1 | |  | Model 2 | |  | Model 3 | |
| --- | --- | --- | --- | --- | --- | --- | --- | --- |
|  | OR (95%CI) | *P* value |  | OR (95% CI) | *P* value |  | OR (95% CI) | *P* value |
| NHHR |  |  |  |  |  |  |  |  |
| Q1 (0.54-2.15] | Ref |  |  | Ref |  |  | Ref |  |
| Q2 (2.15-2.94] | 1.44 (0.76,1.71) | 0.52 |  | 1.11 (0.74,1.65) | 0.62 |  | 1.05 (0.70,1.57) | 0.83 |
| Q3 (2.94-3.95] | 1.02 (0.71,1.47) | 0.92 |  | 0.98 (0.68,1.42) | 0.91 |  | 0.88 (0.60,1.30) | 0.51 |
| Q4(3.95-15.52] | 1.52 (1.12,2.06) | **0.01** |  | 1.42 (1.03,1.95) | **0.03** |  | 1.17 (0.84,1.64) | 0.33 |
| *P* for trend | **0.018** |  |  | 0.067 |  |  | 0.34 |  |

Model 1: Adjusted for no covariates.

Model 2: Adjusted for age, race, education, gender, and *RIP (*ratio of family income to poverty)

Model 3: Adjust for the variables in Model 2 plus BMI (body mass index), moderate recreational activity, smoking, alcohol drinking, hypertension, diabetes

*OR* odd ratio

**Table S2.** Relationship between Hyperlipidemia and kidney stones in the logistic regression models from the NHANES, 2007–2018

|  | Model 1 | |  | Model 2 | |  | Model 3 | |
| --- | --- | --- | --- | --- | --- | --- | --- | --- |
|  | OR (95%CI) | *P* value |  | OR (95% CI) | *P* value |  | OR (95% CI) | *P* value |
| Hyperlipidema |  |  |  |  |  |  |  |  |
| No | Ref |  |  | Ref |  |  | Ref |  |
| Yes | 1.65 (1.46,1.87) | <0.0001 |  | 1.32 (1.15,1.50) | <0.0001 |  | 1.10 (0.95,1.27) | 0.19 |

Model 1: Adjusted for no covariates.

Model 2: Adjusted for age, race, education, gender, and *RIP (*ratio of family income to poverty)

Model 3: Adjust for the variables in Model 2 plus BMI (body mass index), moderate recreational activity, smoking, alcohol drinking, hypertension, diabetes

*OR* odd ratio

Table S3 Result of subgroup analysis based on the presence or absence of metabolic syndrome

| Subgroup | Quartiles of NHHR | | | |
| --- | --- | --- | --- | --- |
|  | Q1 | Q2 | Q3 | Q4 |
| Metabolic syndrome |  |  |  |  |
| No | 1 | 1.28 (1.03,1.58)* | 1.34 (1.06,1.70)* | 1.32 (1.00,1.74)* |
| Yes | 1 | 1.21 (0.86,1.71) | 1.22 (0.83,1.80) | 1.12 (0.78,1.61) |
